# Obesity-Associated Inflammation: Does Curcumin Exert a Beneficial Role?

**DOI:** 10.3390/nu13031021

**Published:** 2021-03-22

**Authors:** Rosaria Varì, Beatrice Scazzocchio, Annalisa Silenzi, Claudio Giovannini, Roberta Masella

**Affiliations:** Center for Gender-Specific Medicine, Gender Specific Prevention and Health Unit, Istituto Superiore di Sanità, Viale Regina Elena 299, 00161 Rome, Italy; rosaria.vari@iss.it (R.V.); beatrice.scazzocchio@iss.it (B.S.); annalisa.silenzi@iss.it (A.S.); claudio.giovannini@iss.it (C.G.)

**Keywords:** curcumin, obesity, inflammation, adipose tissue

## Abstract

Curcumin is a lipophilic polyphenol, isolated from the plant turmeric of Curcuma longa. Curcuma longa has always been used in traditional medicine in Asian countries because it is believed to have numerous health benefits. Nowadays it is widely used as spice component and in emerging nutraceutical food worldwide. Numerous studies have shown that curcumin possesses, among others, potential anti-inflammatory properties. Obesity represents a main risk factor for several chronic diseases, including type 2 diabetes, cardiovascular disease, and some types of cancer. The establishment of a low-grade chronic inflammation, both systemically and locally in adipose tissue, occurring in obesity most likely represents a main factor in the pathogenesis of chronic diseases. The molecular mechanisms responsible for the onset of the obesity-associated inflammation are different from those involved in the classic inflammatory response caused by infections and involves different signaling pathways. The inflammatory process in obese people is triggered by an inadequate intake of nutrients that produces quantitative and qualitative alterations of adipose tissue lipid content, as well as of various molecules that act as endogenous ligands to activate immune cells. In particular, dysfunctional adipocytes secrete inflammatory cytokines and chemokines, the adipocytokines, able to recruit immune cells into adipose tissue, amplifying the inflammatory response also at systemic level. This review summarizes the most recent studies focused at elucidating the molecular targets of curcumin activity responsible for its anti-inflammatory properties in obesity-associated inflammation and related pathologies.

## 1. Introduction

Curcumin, the main natural polyphenol found in the rhizome of Curcuma longa (turmeric) [1], has been recognized for thousands of years because of its medicinal properties and potential health benefits [2]. It is used worldwide in different forms: as spice, antiseptic, anti-inflammatory, preservative or coloring agent, as well as supplement in capsules or powder form [3]. It has been reported the beneficial effect of curcumin in various diseases, including inflammatory and degenerative conditions, cancer, dyslipidemia, metabolic syndrome (MetS), and obesity [4,5,6,7]. Several studies have also shown that most of the benefits are due to its antioxidant and anti-inflammatory activities [5]. Overweight and obesity are a major public health problem all over the world [8]. Obesity is caused by the imbalance between energy intake and energy expenditure, culminating in the excess of fat accumulation in the adipose tissue (AT) [9]. It is associated with a chronic low-grade inflammation that might represent the main factor linking obesity and the development and progression of various diseases including type 2 diabetes (T2D), dyslipidemia, heart diseases, stroke, and cancer [10,11]. AT, indeed, is recognized as an endocrine organ that secretes a number of cytokines and chemokines with regulatory and immune functions [12]. Dysfunctions of the secretory activity of AT, thus, most likely play a pathogenic role in the occurrence of the obesity-related pathologies [13]. In consideration of this, the current review examines specifically the possible role of curcumin in counteracting the activation of inflammatory pathways in AT. To this purpose, we conducted a comprehensive literature search until December 2020 in PubMed, using “obesity”, “inflammation”, “adipose tissue”, “adipocyte” as key words in combination with “curcumin” and “dietary curcumin”.

## 2. Obesity, AT Dysfunction and Inflammation

Obesity is characterized by an excessive AT expansion due to hyperplasia (increase in number) and/or hypertrophy (increase in size) of adipocytes, the major cellular component of AT. Although the main function of adipocytes is the storage and release of lipids, they secrete also active molecules that are used for intracellular signaling and to communicate with every organ system in the body. The second largest AT cellular component beyond adipocytes are resident immune cells that, in turn, play important roles in the maintenance of AT homeostasis. The intensity and complexity of these signal networks are highly regulated, differ in each fat pad, and are dramatically affected by various disease states. In conclusion, AT is an active endocrine organ, secreting a variety of hormones and metabolites that regulate systemic metabolism. When the imbalance in the storage of lipids by fat cells is established, alterations in secretive function occur and systemic metabolic dysfunctions might happen, such as in T2D, cardiovascular and liver diseases, and cancers. Through these cellular derangement and metabolic dysfunction, an excessive caloric intake contributes to a chronic low-grade inflammation, also known as ‘metainflammation’. In particular, visceral AT accumulated in the abdominal seat, shows a disrupted balance between secreted pro- and anti-inflammatory factors, with increased levels of pro-inflammatory adipocytokines, including leptin [14], and decrease of anti-inflammatory adipokines, such as adiponectin [15]. These events all together cause local alterations of the AT environment and alter the normal cross-talk with other organs, such as liver, muscle, brain, and pancreas [16,17], which leads, as a further result, to metabolic dysfunctions, such as hyperinsulinemia and insulin resistance (IR) [11]. Furthermore, the polarization profile of the resident immune cells depends on the health status of the adipocytes [18]. Changes in the adipocyte secretion profile, in fact, trigger the recruitment and activation of immune cells [19]. In particular, in obese subjects, AT macrophages shift from an anti-inflammatory profile (such as that found in normal weight people) towards a pro-inflammatory phenotype [20,21] producing themselves an alteration in the production/activation of key factors that exacerbate local and systemic inflammation [22], such as tumor necrosis factor (TNF)α, interleukin (IL)-6, IL-1β, toll-like receptor (TLR) 4, and nuclear factor (NF)-κB, that may amplify the inflammatory state and favor the onset of pathologies [23,24,25,26,27]. The pro-inflammatory profile in obese individuals is evidenced by elevated serum levels of TNFα and IL-6, simultaneously with adiponectin and anti-inflammatory cytokines decrease [28,29]. The expression of the pro-inflammatory cytokines is regulated by the activation of the transcription factor NF-κB. This factor is stored in the cytoplasm as inactive form bound to the inhibitor IκBα that is, in turn, regulated by the inhibitor of κB kinase (IKK) complex consisting of 2 subunits, IKKα and IKKβ. Different stimuli, including growth factors, cytokines and foreign pathogens or molecules, such as lipopolysaccharides (LPS) and free fatty acids (FFA) [30], activate the IKK kinase complex inducing proteasomal degradation of IκBα and leading to the translocation of NF-κB in the nucleus, where it induces the expression of genes of various inflammatory mediators. Obese people show an increased activation of NF-κB pathway, most likely responsible for the increased pro-inflammatory cytokine release [31]. Among the pro-inflammatory compounds, it should be mentioned leptin; it is primarily produced by AT, its level increases in obese people and participates in the control of body weight by regulating food intake and energy expenditure [32]. On the other hand, adiponectin, produced almost exclusively by AT, circulates in high concentration in plasma and has anti-inflammatory properties probably related to the inhibition of NF-κB activation and, consequently, to the reduced synthesis of pro-inflammatory cytokine [33,34]. The secretion of anti-inflammatory adipocytokines is inhibited in visceral AT from obese patients and subjects with MetS leading to a significant reduction in their plasma levels [35,36]. Despite the intense experimental work carried out, the exact molecular mechanisms responsible for the chronic low-grade metabolic inflammation in obesity are not completely clarified yet. However, with the identification of the nod-like receptor pyrin domain-containing (NLRP)3 inflammasome in AT, a new hypothesis has been formulated suggesting that it might be relevant for regulating obesity-associated inflammation and insulin sensitivity [37]. The NLRP3 inflammasome is a cytosolic molecular complex whose expression in AT directly correlates with body weight and aging, while its inactivation significantly mitigates metabolic disorders [38,39]. A number of exogenous and endogenous signals might act as NLRP3 inflammasome activator in AT leading to the production of pro-inflammatory cytokines [40,41] a potential mechanism linking an elevated intake of saturated fatty acids (SFAs) to the progression of metabolic diseases. In line with this, increased gene expressions of NLRP3 and its key effectors IL-1β and IL-18 have been observed in visceral fat of metabolically unhealthy individuals compared to those from lean healthy control or metabolically healthy obese individuals [42]. Furthermore, these inflammatory effects were suppressed, after weight loss, in the subcutaneous fat of patients with obesity and T2D, with consequent improvement in insulin sensitivity [38]. Studies have hypothesized a causal nexus between systemic inflammation and an increased release of FFA from AT in obese and insulin-resistant subjects [43,44]. Indeed, the direct drainage of free FA and adipokines from visceral AT to the liver can activate immune responses leading to the secretion of inflammatory compounds [17]. A potential mechanism of action through which FFA, and mainly dietary SFAs, can mediate AT dysfunctions contributing to the onset of inflammation involves the TLR4 [45,46]. TLR4 belongs to the TLR family and is expressed not only on leukocytes but also on many non-immune cells, including adipocytes, hepatocytes, and muscle cells. It has been hypothesized that FFA can bind and stimulate TLR4; thus, the elevated plasma level of FFA observed in obesity could activate TLR4. A recent research showed that the TLR4 activation can mediate inflammatory processes also through the impairment of adipogenesis which, in turn, elicit adipocyte and resident immune cell dysfunctions [47]. In conclusion, the onset of inflammatory processes linked to obesity and metabolic dysfunction in AT involves a number of different factors closely intertwined. The inflammation associated with obesity has been shown to derive from changes in the delicate crosstalk between adipocytes and macrophages due to an increased infiltration of macrophages into AT, the activation of a number of pro-inflammatory pathways, the alterations of adipokine production and increased expression and release of a panel of inflammatory cytokines. Understanding the molecular and metabolic switches that, starting from AT, lead to immune cells polarization towards inflammatory phenotypes may allow the definition of interventions capable of leading to the resolution of inflammation and blocking the sequence of events responsible for the occurrence of clinical complications in obesity. Targeting the key intracellular pathways underlying AT dysfunctions might represent a useful tool in counteracting obesity-related pathologies. From this point of view, the identification of potential protective activity of curcumin in positively modulating AT pro/anti-inflammatory balance has been gaining significant interest.

## 3. Curcumin and Inflammation in Obesity

Several studies carried out in humans have shown that curcumin attenuates inflammation in obesity and obesity-related diseases by rebalancing the equilibrium between anti- and pro-inflammatory factors via different mechanisms due to the interactions of curcumin with a wide range of biomolecules, such as transcription factors, cellular receptors, growth factors, enzymes, cytokines, and chemokines [48,49]. Moreover, some reports have suggested that curcumin can enhance weight loss induced by diet and lifestyle intervention on overweight subjects with MetS ([50,51]. However, it should be considered that a main problem in the use of curcumin is its poor bioavailability. To increase curcumin bioavailability, different delivery systems including micelles, liposomes, phospholipid complexes, nanostructured lipid carriers, and biopolymer nanoparticles have been developed, as well as the addition of piperine, a bioactive alkaloid extracted from the Piper species, which has been shown to effectively enhance the bioavailability of several nutritional supplements including curcumin [52].

## 4. Curcumin Decreases Circulating Inflammatory Markers in Overweight/Obese Subjects

There is an increasing evidence that curcumin treatment could be able to alleviate the altered pro-inflammatory mediator secretions present in obesity and related pathologies. In this section, data from human studies carried out on overweight and obese subjects with curcumin supplementation are collected and summarized. A research performed on 84 overweight or obese patients with non-alcoholic fatty liver disease (NAFLD) demonstrated that, curcumin supplementation with two 40 mg capsules/day after meals for 3 months, induces a decrease in many serum inflammatory markers, such as TNFα, high-sensitive C-reactive protein (hs-CRP), and IL-6 [53]. The same conclusions were reached by other studies carried out in obese/overweight people; specifically, curcumin administration (1 g/day) for 8 weeks reduces serum concentrations of TNFα, IL-6, and monocyte chemoattractant protein 1 (MCP-1) in males and females with diagnosis of MetS with respect to the placebo group [7]. In a randomized placebo-controlled clinical trial carried out on 60 adolescent girls undergoing to a slight weight-loss diet for 10 weeks, curcumin consumption (500 mg/day) was able to induce a significant decrease in hs-CRP and IL-6 compared to placebo supplementation [54]. In addition, it has been demonstrated that curcumin modulates circulating levels of IL-1β in thirty subjects randomized to receive curcumin (1 g/day) or a matched placebo for 4 weeks. Serum IL-1β was found to be significantly reduced by curcumin treatment. In contrast, no significant difference was observed in the concentrations of IL-6, and MCP-1 [55]. Finally, curcuminoids supplementation (300 mg/day) for 3 months in T2D patients led to a significant decrease in circulating FFA levels [56], that are considered a major factor linking obesity and inflammation [57,58,59]. 

## 5. Curcumin Modulates Adipokines

Adiponectin and leptin are two important adipokines released by adipocytes that have [18] several target organs including brain, liver, pancreas, muscle, immune system, and AT itself. They are involved in inflammation and immune response, showing, as stated above, adiponectin anti-inflammatory properties, leptin, on the contrary, pro-inflammatory ones [60]. Obese subjects are characterized by an imbalance of the two adipokines showing a low concentration of adiponectin and high levels of leptin in plasma [61]. Curcumin has been shown to increase the production of adiponectin [62]. To this regard, a systematic review [63] showed that curcuminoid administration significantly increased plasma adiponectin concentrations in randomized controlled trials. Specifically, in a double-blind randomized trial carried out over a 12-week period on 118 patients with T2D the effects of the daily administration of 1 g curcumin added with 10 mg piperine were compared to placebo. The treatment with curcumin plus piperine reduced serum levels of TNFα and increased serum level of adiponectin [64]. In another study, curcumin supplementation (1 g/day) for 6 weeks increased serum adiponectin concentrations compared to both curcumin-phospholipid complex (1 g/day) and placebo groups in 120 men and women with MetS [65]. In a randomized double-blind study 44 men and women with T2D were treated with curcumin 1500 mg/day or placebo for 10 weeks. At the end of the study, a significant increase in serum adiponectin concentration together with a decrease in the mean weight were observed in the curcumin group [66]. Conversely, no effect on adiponectin was seen in 22 young men randomly assigned to receive curcumin (500 mg/day) or placebo for 12 weeks. This finding might be determined by the low dose of curcumin used for the treatment [67]. However, the same amount of curcumin (500 mg/day) for 4 weeks reduced serum leptin and resistin and increased adiponectin content in 15 children and 15 adults [68,69]. Accordingly, elevated levels of adiponectin and decreased leptin levels were reported in diabetic men and women after 6-month intervention with a high dose of curcumin (1500 mg/day) [70]. Similar effects on serum levels of leptin were observed in males and females with NAFLD treated for 12 weeks with even higher doses of curcumin (3000 mg/day) [71]. In conclusion, all the studies discussed show that curcumin supplementation contributes to rebalance pro- and anti-inflammatory factor production significantly increasing the levels of anti-inflammatory adipocytokines, such as adiponectin, and decreasing the pro-inflammatory ones, such as TNFα, IL-6, IL-1β, and MCP-1, counteracting the chronic inflammatory condition in overweight/obese subjects (Table 1). 

## 6. Effects of Curcumin on Inflammatory Signaling Pathways 

**Figure 1:**
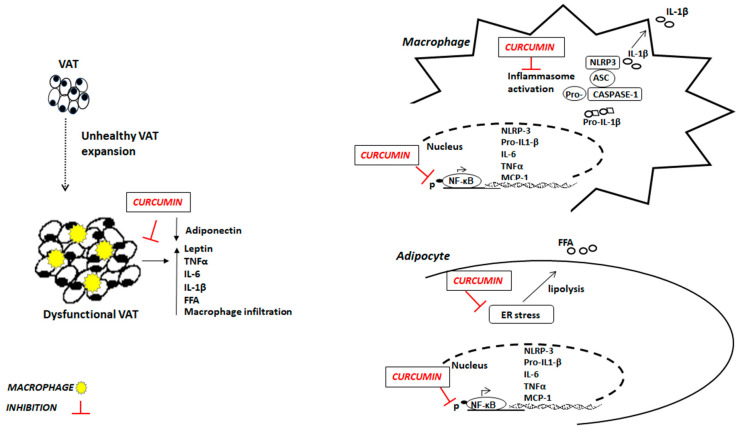
Potential anti-inflammatory mechanisms of curcumin in obesity. VAT: visceral adipose tissue; FFA: free fatty acids; TNFα: tumor necrosis factor α; IL-6: interleukin 6; IL-1β: interleukin β; ER: endoplasmic reticulum; NF-κB: Nuclear transcriptor factor kappa B; MCP-1: Monocyte chemoattractant protein-1; NLRP-3: nod-like receptor pyrin domain-containing 3.

Most of the potential molecular mechanisms responsible for the health effects of curcumin have been studied in animals and in vitro models using stabilized cell lines or human primary cells. Although the results obtained in this way cannot be completely extrapolated to humans, they allowed to suggest possible mechanisms of curcumin action that could explain the phenotypic effects evidenced by the studies carried out in humans (Figure 1). As regards animal studies, the anti-inflammatory effect of curcumin was first demonstrated in acute and chronic models of inflammation in rats and mice [72]. Specifically, in obese mice, curcumin treatment (3% by weight for 6 weeks) decreases NF-κB activity in liver tissue, associated with decreased hepatic expression of inflammatory molecules, such as TNFα and MCP-1. Curcumin-treated obese mice also show a decreased macrophage infiltration and an increased expression of forkhead transcription factor (Foxo)1 and adiponectin into AT, and higher circulating adiponectin levels [72]. In line with these results, an in vivo study performed in male rats, T2D insulin resistant because of high-fat diet (HFD) consumption, demonstrated oral administration of curcumin (80 mg/kg body weight) was able to improve insulin sensitivity by attenuating TNFα serum levels [73]. Moreover, dietary curcumin (4 g/kg diet added 2 days/week) attenuated the inflammatory response induced by HFD in mice through inhibiting NF-κB expression and JNK signaling pathway in epididymal AT [74]. Interestingly, administration of 0.1% curcumin associated with white pepper (0.01%) (Curcuma-P^®^) significantly down-regulated the proinflammatory cytokines IL-6 and TNFα, but did not modify IL-1β and MCP-1, in the subcutaneous AT of mice after 4 weeks of HFD. This effect was relatively tissue-specific and independent on macrophage infiltration. Indeed, the inflammatory cell infiltration in AT was not modified by Curcuma-P^®^ supplementation [75]. Another interesting activity of curcumin has been demonstrated on endoplasmic reticulum (ER). The involvement of ER stress (with accumulation of misfolded proteins) in the release of FFA has been observed in several studies [76,77,78]. Curcumin has been demonstrated to mitigate ER stress in mice fed HFD and in primary adipocytes. Specifically, short-term HFD feeding (10 days) increased ER stress in mouse AT by increasing the expression of phospho-inositol-requiring kinase 1(p-IRE1) and phospho-eukaryotic Initiation Factor 2 (p-eIF2), two important indicators of ER stress. Oral administration of curcumin (50 mg/kg) counteracted the activation of IRE1 and eIF2 by reducing the phosphorylation, and consequently inhibiting the ER stress in vivo. Similarly, curcumin (0.1, 1, 10 µM) treatment inhibited IRE1 and eIF2 activation in mouse AT treated with 100 µM of palmitate (inductor of ER stress). Furthermore, curcumin administration reduced glycerol and FFA released from AT of HFD-fed mice blocking cAMP/PKA signaling via regulation of AMP-activated protein kinase (AMPK) [79], as well as significantly decreased plasma FFA levels in HF-induced obese rats [80]. Several in vitro models have been used to collect more detailed information about the potential molecular mechanisms of action through which curcumin exerts its effects. Curcumin has been shown to inhibit the activation of the pro-inflammatory NF-κB signaling pathway in several cell types, including human adipocytes and macrophages [48]. In adipocytes treated with TNFα to induce inflammatory processes, the contemporary treatment with 20 μM curcumin suppressed the degradation of IκBα, the NF-κB inhibitor, reducing, consequently, NF-κB translocation to the nucleus and significantly inhibiting the expression of TNFα, IL-1β, IL-6 and COX2 genes and IL-6 secretion [81]. In the same type of cells, curcumin also exerts a protective effect on hypoxia in a dose-dependent manner (5, 10, and 20 µM) reducing the secretion of the inflammatory cytokines and protecting mitochondrial functions [82]. Besides a direct effect on adipocytes, curcumin has been shown to exert anti-inflammatory effects by counteracting the increased recruitment of macrophages in AT from obese mice [72,83]. Several studies have evidenced that curcumin treatment reduces macrophage invasion of AT in mouse models of obesity [62,72]. It has been shown that the cross-talk between adipocytes and macrophages in AT triggers and increases inflammatory responses in obesity including the increased production of MCP-1 and other inflammatory cytokines [84,85]. Curcumin treatment (0.1–10 µM) of Raw 264.7 macrophages incubated with the culture medium of mesenteric AT taken from obese mice, potentially able to induce an inflammatory response, significantly inhibited the production of TNFα, MCP-1, and nitric oxide, as well as the migration capacity of the macrophages with respect to the cells not treated with curcumin. Furthermore, 10 µM curcumin treatment significantly inhibited MCP-1 release from 3T3-L1 adipocytes [86]. Studies carried out in different cell systems strongly suggest that the anti-inflammatory activity of curcumin occurs by modulating NLRP3 inflammasome. In THP-1 macrophages treated with phorbol 12-myristate 13-acetate (PMA), an activator of NLRP3 inflammasome, curcumin (6.25, 12.5, and 25 μM) reduced NLRP3 inflammasome level, the activation of caspase-1 and the secretion of IL-1β in a dose-dependent manner, most likely down-regulating TLR4/NF-κB signal transduction pathway that is involved in NLRP3 inflammasome activation [87]. In mouse bone marrow-derived macrophages (BMDM) treated with nigericin, another NLRP3 inflammasome activator, the pre-treatment with curcumin (30–50 μM) inhibited caspase-1 cleavage and IL-1β secretion. The same results were observed in human macrophages. Specifically, differentiated THP-1 cells pretreated with curcumin showed reduced caspase-1 activation and IL-1β secretion after treatment with LPS and nigericin. The inhibition of NLRP3 activation by curcumin appears to be due to the suppression of K^+^ efflux [88].

In conclusion, a growing body of experimental data supports the hypothesis that the beneficial effects of curcumin on obesity-related pathologies may be related to the suppression of IL-6, TNFα, IL-1β, and MCP-1 expression from adipocytes, the inhibition of macrophage recruitment in AT, and the inhibition of the inflammatory activity of the NLRP3 inflammasome [89] (Table 2).

## 7. Conclusions

The inflammation present in AT is involved in the development of various obesity-related pathologies. The studies reported in this review clearly show that curcumin supplementation significantly decreases inflammatory cytokine production and increases adiponectin level in plasma of obese and overweight subjects. Furthermore, curcumin can regulate several molecular targets including transcription factors (NF-kB, NLP3), signaling pathways and other complex regulatory systems in AT resulting in the suppression/attenuation of the chronic low-grade inflammation. However, since curcumin is widely used as a supplement around the world because of its health promoting properties, further studies, both in vitro to better define the mechanisms of action, and in humans by controlled gender-based clinical trials to evaluate the real effectiveness, are mandatory. It should be reached, thus, that ultimate evidence on curcumin effects and highlighted possible differences in the response to curcumin treatment between women and men, allowing the definition of personalized advice about curcumin consumption.

## Figures and Tables

**Table 1:** Effects of curcumin on inflammation in obesity: human studies.

Study Design	Subjects	Treatment	Duration	Outcomes	References
Randomized double-blind, placebo-controlled	Overweight/obese with NAFLD (males and females, *n* = 84)	42 curcumin (40 mg/day) 42 placebo	3 months	↓TNF-alpha and IL-6	[53]
Randomized, double-blind, placebo-controlled	Overweight/obese with MetS (males and females, *n* = 117)	59 curcumin (1 g/day) 58 placebo	8 weeks	↓TNF-α, IL-6, and MCP-1	[7]
Randomized, double-blind, placebo-controlled	Overweight/obese (adolescent girls, *n* = 60)	30 curcumin (500 mg/day) 30 placebo	10 weeks	↓IL-6	[54]
Randomized, double blind, crossover	Obese (males and females, *n* = 30)	15 curcumin (1g/day + 5 mg bioperine) 15 placebo	4 weeks each treatment + 2 weeks wash-out between the regimens.	↓IL-1β no changes IL-6, and MCP-1	[55]
Randomized, double-blind, placebo-controlled	Overweight/obese with T2D (males and females, *n* = 100)	50 curcumin (300 mg/day) 50 placebo	3 months	↓FFA	[56]
Randomized, double-blind, placebo-controlled	T2D (unspecified gender *n*= 100)	50 curcumin (1 g + 10 mg piperine/day) 50 placebo	12 weeks	↓TNF-α and Leptin ↑ Adiponectin	[64]
Randomized, double-blind, placebo-controlled	Overweight with T2D (males and females, *n* = 44)	21 curcumin (1500 mg/day) 23 placebo	10 weeks	↑ Adiponectin ↓weight	[66]
Randomized, double-blind, placebo-controlled	Obese (males and females, 29 adults, 29 children)	15 children curcumin (500 mg/day) 14 children placebo 15 adults curcumin (500 mg/day) 14 adults placebo	4 weeks	↓Leptin ↓Resistin ↑Adiponectin	[68,69]
Randomized, double-blind, placebo-controlled	Obese with MetS (males and females, *n* = 120)	40 curcumin (1 g/day) 40 placebo 40 phospholipidated curcumin (1 g/day)	6 weeks	↑ Adiponectin	[65]
Randomized, double-blind, placebo-controlled	Overweight T2D (males and females, *n* = 210)	107 curcumin (1.5 g/day) 103 placebo	6 months	↓ Leptin ↑ Adiponectin	[70]
Randomized double-blind, placebo-controlled	Overweight\obese with NAFLD (males and females, *n* = 46)	23 curcumin (3 g/day) 23 placebo	12 weeks	↓Leptin	[71]
Randomized double-blind, placebo-controlled	Obese (males, *n* = 22)	11 curcumin (500 mg/day) 11 placebo	12 weeks	no change Adiponectin	[67]

Abbreviations: ↑ Increases; ↓ Decreases; IL-6, interleukin-6; IL-1β, interleukin-1β; MCP-1, monocyte chemoattractant protein-1; TNFα, tumor necrosis factor α; FFA, free fatty acids; T2D, type 2 diabetes; MetS, metabolic syndrome; NAFLD; nonalcoholic fatty liver disease.

**Table 2:** Effects of curcumin on inflammation in obesity: in vivo and in vitro studies.

Animal Model	Diet	Duration	Outcome	References
Male C57BL/6 mice Wild-type and ob/ob	Standard diet (4% fat) ± curcumin 3% by weight HFD (35% fat) ± curcumin 3% by weight (*n* = 5/group)	6-weeks	in adipose tissue ↑Foxo1 and adiponectin expression ↓infiltration of macrophages ↑circulating Adiponectin levels ↓MCP-1. ↓TNFα, MCP-1 expression, and NF-κB activity in liver	[72]
Male Sprague Dawley rats	Standard diet (control) HFD HFD + curcumin (80 mg/kg/day) (*n* = 11/group)	60 days 75 days	↓FFA and TNFα serum levels in all group compared to non-treated HFD groups	[73]
Male C57BL/6J mice	Low-fat diet (10% Kcal from fat) HFD (45% Kcal from fat) HFD + curcumin (4 g/kg diet) (*n* = 12/group)	28 weeks	in adipose tissue ↓macrophage infiltration ↓NF-κB expression and JNK signaling pathway activation	[74]
Male C57BL/6J mice	Standard Diet (control) HFD HFD + Curcuma-P^®^ (0.1% curcumin + 0.01% white pepper) (*n* = 8/group)	4 weeks	↓IL-6 and TNFα, no changes in MCP1, IL-1β, CD68, and F4/80 in adipose tissue	[75]
Male C57BL/6 mice	HFD HFD + curcumin (50 mg/kg) (*n* = 6/group)	10 days	↓ER stress in adipose tissue ↓FFA release from adipose tissue	[79]
Male Wistar rats	Standard diet (control) HFD HFD + curcuminoid (30, 60, 90 mg\Kg body weigth\day) (*n* = 12/group)	12 weeks	↓FFA plasma levels	[80]
**Cell type**				
Raw 264.7 macrophages treated with conditioned medium by mesenteric adipose tissue 3T3-L1 adipocytes	0.1–1–10 µM curcumin 10 µM curcumin	24 h	↓TNFα and MCP-1 release ↓MCP-1 release	[86]
3T3-L1 adipocytes treated with TNF-α	2–20 μM curcumin	62 h	↓NF-κB activation. ↓TNFα, IL-1β, IL-6, expression ↓IL-6 secretion	[81]
3T3-L1 adipocytes 24-h hypoxia	5, 10, 20 mM curcumin	24 h	↓TNFα, IL-1β, IL-6 release	[82]
THP-1 macrophages treated with PMA	6.25, 12.5, 25 μM curcumin	24 h	↓NLRP3 inflammasome expression, caspase-1 activation, IL-1β secretion, TLR4 expression, and NF-κB activation	[87]
Mouse, bone marrow-derived macrophages (BMDM) treated with nigericin (10 mM) THP-1 cells treated with LPS	30–50 μM curcumin 30–50 μM curcumin	1 h	↓caspase-1 cleavage ↓IL-1β secretion ↓caspase-1 activation ↓IL-1β secretion	[88]

Abbreviations: ↑ Increases; ↓ Decreases; IL-6, interleukin-6; IL-1β, interleukin-1β; MCP-1, monocyte chemoattractant protein-1; TNFα, tumor necrosis factor α; FFA, free fatty acids; HFD, high-fat diet; ER, endoplasmic reticulum; LPS, lipopolysaccharides; PMA, phorbol 12-myristate 13-acetate; Foxo1, forkhead transcription factor 1; NF-κB, nuclear transcriptor factor kappa B; JNK, jun N-terminal kinase; TLR4, toll-like receptor 4; NLRP3, nod-like receptor pyrin domain-containing 3.

## Data Availability

Data sharing is not applicable to this article.
